# Real-World Tolerability and Effectiveness of Nanoliposomal Irinotecan in Metastatic Pancreatic Cancer: A Multicentre Indian Experience

**DOI:** 10.7759/cureus.117689

**Published:** 2026-10-09

**Authors:** Chandrakanth MV, Sushant Mittal, Chandragouda Dodagoudar, Chetna Bakshi

**Affiliations:** 1 Oncology, Narayan Multispeciality Hospital, Kolkata, IND; 2 Medical Oncology, Balaji Action Cancer Hospital, New Delhi, IND; 3 Medical Oncology, BLK-Max Super-Specialty Hospital, New Delhi, IND; 4 Medical Oncology, Jupiter Hospital, Mumbai, IND

**Keywords:** metastatic pancreatic ductal adenocarcinoma, nal-iri, nanoliposomal irinotecan, overall survival, real-world evidence, tolerability

## Abstract

Background

Metastatic pancreatic ductal adenocarcinoma (mPDAC) is associated with poor prognosis and has limited therapeutic options. Nanoliposomal irinotecan (Nal-IRI), evaluated in the NAPOLI-1 and NAPOLI-3 clinical trials, has demonstrated survival benefit in later-line and first-line settings, respectively. However, real-world data, particularly from low- and middle-income countries (LMICs) such as India, remain limited. We aimed to evaluate the real-world tolerability and early effectiveness of nal-IRI-based regimens in Indian patients with mPDAC.

Methods

This multicentre, retrospective observational study included 24 patients with histologically confirmed mPDAC treated with nal-IRI-based regimens (either nal-IRI + 5-FU/LV or nal-IRI + 5-FU/LV + oxaliplatin) between June 2024 and September 2025. The primary objective was to assess tolerability using CTCAE v5.0-graded adverse events (AEs). Secondary objectives included early effectiveness outcomes, assessed by overall survival (OS) using the Kaplan-Meier method. Restricted mean survival time (RMST) was calculated at three- and six-month landmarks due to limited follow-up.

Results

The mean age was 63.7 years (SD 6.89), and 75% of patients were male. Nal-IRI was administered in the first-line setting in six patients and in second or later lines in 18 patients. The median follow-up was four months. A total of 55 AEs were reported, most commonly diarrhea (20%), fatigue (12.7%), and mucositis and neutropenia (10.9% each). Among graded events, 26.4% were grade 1, 39.6% were grade 2, and 34.0% were grade 3. Dose modifications were required in 36.5% of events, and treatment discontinuation due to AEs occurred in 11.5% of events. Kaplan-Meier-estimated OS probabilities at three and six months were 95% and 88%, respectively, with an RMST of 2.89 months up to three months and 5.57 months up to six months.

Conclusions

In this multicentre real-world Indian cohort, nal-IRI-based regimens demonstrated manageable tolerability. These findings support the feasibility of nal-IRI in routine clinical practice and provide important real-world evidence (RWE) from an underrepresented population. However, survival outcomes were exploratory because of the short follow-up and high degree of censoring. Larger prospective studies with longer follow-up are warranted.

## Introduction

Pancreatic cancer is a rare malignancy in India, with approximately 13,661 new cases and 12,759 deaths reported annually [1]. The significant mortality burden underscores a substantial unmet need for novel formulations and targeted therapies with more acceptable tolerability profiles.

The current standard of care for metastatic pancreatic ductal adenocarcinoma (mPDAC) is chemotherapy. Nanoliposomal irinotecan (nal-IRI) is a liposomal formulation of irinotecan that prolongs systemic exposure to irinotecan and enhances tumor tissue penetration of SN38 (the active metabolite of irinotecan). This potentially improves therapeutic efficacy while reducing off-target effects [2,3]. Nal-IRI combined with 5-fluorouracil and leucovorin (5-FU/LV) demonstrated a significant survival benefit compared with 5-FU/LV alone in gemcitabine- plus nab-paclitaxel-pretreated patients with mPDAC in the pivotal phase III NAPOLI-1 trial [3-6]. The phase III NAPOLI-3 trial showed that a first-line regimen of nal-IRI + 5-FU/LV + oxaliplatin (NALIRIFOX) significantly improved survival outcomes compared with the standard nab-paclitaxel + gemcitabine regimen in patients with untreated mPDAC [7]. 

The National Comprehensive Cancer Network (NCCN) recommends nal-IRI-based regimens for mPDAC, including NALIRIFOX in the first-line setting and nal-IRI + 5-FU/LV in the second- or later-line settings [8]. Nal-IRI with 5-FU/LV is an approved regimen for second-line metastatic pancreatic cancer in India by the Central Drugs Standard Control Organisation (CDSCO).

Although nal-IRI has demonstrated a survival benefit in randomized trials such as NAPOLI-1 and NAPOLI-3 and in global real-world evidence (RWE) studies [2,9-11], evidence from low- and middle-income countries (LMICs) remains limited. Differences in patient characteristics, treatment sequencing, and toxicity management may influence outcomes in routine practice. Therefore, real-world data from Indian patients are important to understand the tolerability and effectiveness of nal-IRI in this population. The primary objective of this study was to evaluate the real-world tolerability of nal-IRI-based regimens, assessed using CTCAE v5.0-graded adverse events (AEs), in patients with mPDAC across first-line and subsequent-line treatment settings. The secondary objective was to evaluate early effectiveness, assessed by overall survival (OS).

## Materials and methods

Study design and patient population 

This was a multicentre, retrospective, real-world study conducted across five tertiary oncology centres in India, comprising private and non-government charitable institutions located in West Bengal, Delhi, and Maharashtra. These centres were selected based on the availability of eligible patient records. The study evaluated the real-world tolerability and effectiveness of nal-IRI-based regimens in patients with mPDAC. Consecutive eligible patients treated with nal-IRI-based regimens between June 1, 2024, and September 30, 2025, were included.

The inclusion criteria were as follows: eligible patients were ≥18 years of age; histologically confirmed pancreatic ductal adenocarcinoma; radiologically confirmed metastatic disease; and exposure to at least one cycle of a nal-IRI-based regimen during the study period.

The exclusion criteria were as follows: non-availability of complete records; pancreatic cancer of non-adenocarcinoma histology.

All data were collected retrospectively from hospital records. Medical records of patients treated with nal-IRI-based regimens during the study period were retrospectively screened at each participating centre. Potentially eligible patients were assessed against the predefined inclusion and exclusion criteria, and eligible patients were included in the analysis. Data were then retrospectively extracted from the available hospital records using standardized case report forms (CRFs). Variables collected included demographics and clinical characteristics, prior treatment history, nal-IRI treatment details, treatment modifications, AEs, and survival outcomes. Data collected from the participating centres were subsequently compiled into the study database for analysis. Independent duplicate data abstraction was not performed. Given the retrospective design, no treatment assignment was protocol-mandated. Treatment regimen (nal-IRI + 5-FU/LV or nal-IRI + 5-FU/LV + oxaliplatin) and line of therapy (first-line or subsequent lines) were determined at the treating physician’s discretion. Doses of 5-FU/LV and oxaliplatin were individualized according to routine clinical practice, with dose modifications as clinically indicated; a uniform protocol-mandated dosing schedule was not used. Clinical and radiological follow-up assessments were performed according to routine clinical practice at the participating centres; no minimum follow-up duration was specified as an eligibility requirement. Patients receiving nal-IRI in either first-line or later-line settings were included.

Ethics approval 

The study was conducted in accordance with the principles of Good Clinical Practice and the Declaration of Helsinki, including applicable amendments (2024 revision), as well as the National Ethical Guidelines for Biomedical and Health Research Involving Human Participants (Indian Council of Medical Research, 2017). The study was submitted to the Ethics Committee under protocol NAL-IRI-2026, version 1.0, dated February 9, 2026. Permission to conduct the study was granted via letter no. NSHEC/INV/NON-REG-2026/002, with a waiver of informed consent due to the retrospective nature of the study and the use of anonymized data.

Statistical analysis 

No formal sample size or power calculation was performed because of the retrospective, descriptive nature of the study; the sample size was determined by the number of eligible patients identified during the study period. Descriptive statistics were used to summarize baseline demographic and clinical characteristics. Continuous variables (e.g., age) were summarized using means and standard deviations or medians and interquartile ranges, as appropriate, while categorical variables were summarized using frequencies and percentages. No imputation was performed for missing data. Available-case analysis was used for descriptive summaries, whereby patients with missing values were excluded only from calculations involving the respective variable; complete-case analysis was used for analyses requiring multiple variables. The number of patients contributing to each analysis is reported where applicable.

Tolerability was assessed based on the frequency and severity of AEs. AEs were retrospectively identified from the available hospital records and graded according to the Common Terminology Criteria for Adverse Events (CTCAE), version 5.0 [12]. All AEs were summarized descriptively. Performance status was reported according to the Eastern Cooperative Oncology Group (ECOG) Performance Status scale [13].

Time-to-event outcomes were estimated using the Kaplan-Meier method. Real-world overall survival (rwOS) was defined as the time from initiation of nal-IRI therapy to death from any cause. Patients who were alive at the last available follow-up were right-censored at the date of their last known follow-up. In the Kaplan-Meier analysis, patients contributed to the risk set until the time of death or censoring. Given the limited follow-up duration and potential immaturity of median survival estimates, three- and six-month time points were selected as fixed, clinically interpretable short-term follow-up horizons to characterize early and intermediate short-term survival within the available observation period. Kaplan-Meier survival probabilities were estimated at these time points, and restricted mean survival time (RMST) was calculated up to three and six months to summarize average survival within these fixed horizons without extrapolation beyond the observed survival period. No adjustment for multiple comparisons was performed, as the analyses were primarily descriptive and exploratory rather than confirmatory.

Descriptive analyses and Kaplan-Meier survival analysis were performed using IBM SPSS Statistics for Windows, Version 26.0 (Released 2018; IBM Corp., Armonk, NY, USA). Landmark survival probabilities at three and six months and RMST estimates up to three and six months were calculated using R version 4.1.1 (R Foundation for Statistical Computing, Vienna, Austria) with the survival package. No SPSS extension or macro was used for RMST estimation.

## Results

Baseline demographics 

Patient Characteristics

Twenty-four patients were enrolled in the study; 75% of patients were male; the mean age (SD) was 63.7 (6.98) years. Of the 24 patients, six (25.0%) received nal-IRI-based therapy in the first-line setting, nine (37.5%) in the second-line setting, and nine (37.5%) in the third-line or later setting. Baseline characteristics are summarized in Table 1. Baseline CA19-9 values were available for 15 patients. The mean CA19-9 level was 456.83 ± 327.47 U/mL, while the median value was 449.0 U/mL (IQR: 234.5-492.5 U/mL). The wide range of values (111.4-1226 U/mL) and relatively large standard deviation indicate substantial variability in baseline tumor burden among patients. The similarity between the mean and median suggests that the distribution was not markedly skewed despite the presence of some patients with very high CA19-9 levels.

**Table 1:** Baseline demographic and clinical characteristics of the study population Values are presented as number/total number (percentage). Percentages are calculated using the denominator indicated for each section. ECOG denotes Eastern Cooperative Oncology Group. ECOG performance status was missing in six patients due to incomplete documentation in the retrospective records. * Comorbidity data were available for 20 patients. Patients may have had more than one comorbidity; therefore, percentages for comorbidities do not total 100%. † Previous line therapy percentages are calculated among patients who received nanoliposomal irinotecan (nal-IRI) beyond the first-line setting (n = 18). ‡ Other regimens included gemcitabine plus capecitabine and paclitaxel plus carboplatin.

Characteristic	No./Total (%)
Age (N = 24)	
<65 years	14/24 (58.33%)
≥65 years	10/24 (41.67%)
Mean	63.7 (SD, 6.98)
Sex (N = 24)	
Male	18/24 (75.00)
Female	6/24 (25.00)
ECOG Performance Status (N = 24)	
0	3/24 (12.5%)
1	10/24 (41.7%)
2	4/24 (16.7%)
3	1/24 (4.2%)
Missing	6/24 (25.00)
CA-19-9 (N = 15)	
Mean ± SD	456.83 ± 327.47
Median (IQR)	449.0 (234.5-492.5)
Range	111.4-1226
Comorbidities (n = 20)*	
Diabetes mellitus	10/20 (50.00)
Hypertension	12/20 (58.30)
Other	3/20 (12.50)
Number of Prior Lines of Chemotherapy (N = 24)	
First-line (0 prior lines) (first-line nal-IRI)	6/24 (25.00)
Second-line (1 prior line)	9/24 (37.50)
Third-line or later (>1 prior line)	9/24 (37.50)
Previous Line Therapy† (n = 18)	
FOLFIRINOX-based	4/18 (22.22)
Gemcitabine-nab-paclitaxel	10/18 (55.56)
Other‡	4/18 (22.22)
Average Number of Cycles	
First-line	4.60
Second-line	4.10

Nal-IRI Dose 

Dose information was available for all 24 patients. The mean nal-IRI dose was 60.3 mg/m² (SD, 24.0). The administered dose ranged up to 120 mg/m².

Primary endpoint 

The median follow-up duration was four months.

Most Common AEs

There were 55 AEs reported during the study period in 24 patients, as mentioned in Table 2.

**Table 2:** Most common adverse events reported during nal-IRI-based therapy N = Events. Adverse events are presented according to treatment regimen. Percentages in each treatment column are calculated using the total number of adverse events within that regimen as the denominator (41 events in the later-line group and 14 events in the first-line group). Percentages in the total column are calculated using all reported adverse events (N = 55). Percentages may not total 100% because of rounding. No grade 4 or grade 5 events were reported. Nal-IRI, nanoliposomal irinotecan

Adverse Event	Total (N = 55); no. (%)	Nal-IRI + 5-FU/LV + Oxaliplatin (First Line) N = 14; no. (%)	Nal-IRI + 5-FU/LV (Later Line) N = 41; no. (%)
Gastrointestinal			
Diarrhea	11 (20.0)	2 (14.3)	9 (22.0)
Mouth ulcer	6 (10.9)	0	6 (14.6)
Abdominal pain	5 (9.1)	1 (7.1)	4 (9.8)
Nausea	3 (5.5)	0	3 (7.3)
Vomiting	2 (3.6)	2 (14.3)	0
Hematologic			
Neutropenia	6 (10.9)	1 (7.1)	5 (12.2)
Anemia	4 (7.3)	0	4 (9.8)
Thrombocytopenia	3 (5.5)	0	3 (7.3)
General or Treatment-Related			
Fatigue	7 (12.7)	3 (21.4)	4 (9.8)
Treatment intolerance	2 (3.6)	1 (7.1)	1 (2.4)
Fever	1 (1.8)	1 (7.1)	0
Ascites	1 (1.8)	1 (7.1)	0
Hepatic			
Transaminitis	1 (1.82)	0	1 (2.44)
Neurologic			
Peripheral neuropathy	2 (3.64)	2 (14.33)	0
Metabolic			
Hyponatremia	1 (1.82)	0	1 (2.44)
Total number of adverse events	55 (100)	14 (25.50)	41 (74.55)

AE Severity (CTCAE Grading)

CTCAE severity grading was available for 53 of 55 AEs (96.4%). Details are provided in Table 3.

**Table 3:** Adverse events according to CTCAE grade Distribution of adverse events according to CTCAE grade. CTCAE grading was available for 53 of 55 reported adverse events (96.4%). Percentages are calculated using the number of graded events (N = 53) as the denominator. No grade 4 or grade 5 events were observed.

CTCAE Grade	No. (%) of Graded Events (N = 53)
Grade 1	14 (26.42)
Grade 2	21 (39.62)
Grade 3	18 (33.96)

Management of AEs

Management actions for AEs were documented for 52 of 55 AEs (94.5%) (Table 4). Most patients (34.55%) were managed with dose modification (dose delay or reduction, with or without granulocyte colony-stimulating factor (G-CSF), including oxaliplatin dose reduction). Only 10.91% of patients discontinued treatment.

**Table 4:** Management of adverse events Management details were available for 52 of 55 reported adverse events (94.5%). Percentages are calculated using events with documented management (N = 52) as the denominator. Percentages may not total 100 because of rounding. * Dose modification included dose delay or dose reduction, with or without granulocyte colony-stimulating factor (G-CSF), including oxaliplatin dose reduction. † Supportive pharmacotherapy included analgesics, antidiarrheal agents, antiemetics, and intravenous fluids. ‡ Supportive care included nonpharmacologic measures such as topical oral care.

Management Strategy	No. (%) of Events With Documented Management (N = 52)
Dose modification*	19 (36.54)
Supportive pharmacotherapy†	14 (26.92)
Supportive care‡	10 (19.23)
Drug discontinuation	6 (11.54)
Observation only	3 (5.87)

At the patient level, dose reduction was documented in nine of 24 patients, while drug discontinuation was documented in eight patients. At the event level, management actions were documented for 52 of 55 AEs (94.5%); among these events, 19 (36.5%) involved dose modification, and six (11.5%) resulted in drug discontinuation.

ECOG Performance Status Among Patients Requiring Dose Reduction or Drug Discontinuation

Among patients who required dose reduction, 22.2% had ECOG 2-3. Among those who discontinued treatment, 37.5% had ECOG 2-3 (Table 5). Although patients with ECOG 2-3 represented only 5/24 (20.8%) of the overall cohort, they accounted for a larger proportion of treatment discontinuations (37.5%), suggesting a possible trend toward poorer treatment tolerance in patients with worse baseline performance status.

**Table 5:** ECOG performance status among patients requiring dose reduction or drug discontinuation ECOG, Eastern Cooperative Oncology Group

Outcome	Missing ECOG, n (%)	ECOG 0-1, n (%)	ECOG 2-3, n (%)	Total
Dose Reduction	2 (22.2)	5 (55.6)	2 (22.2)	9 (100.0)
Drug Discontinuation	2 (25.0)	3 (37.5)	3 (37.5)	8 (100.0)

Secondary endpoint 

Real-World Overall Survival (rwOS)

RMST analysis was performed because the median OS could not be reliably estimated with only two deaths observed and 22 patients (91.7%) censored during follow-up. The RMST was 2.89 months up to three months and 5.57 months up to six months. Given the small number of observed deaths and substantial censoring, these estimates should be interpreted as descriptive measures of short-term survival.

Landmark survival estimates were 95% at three months and 88% at six months, suggesting favorable short-term survival in this cohort (Figure 1 and Table 6).

**Figure 1:**
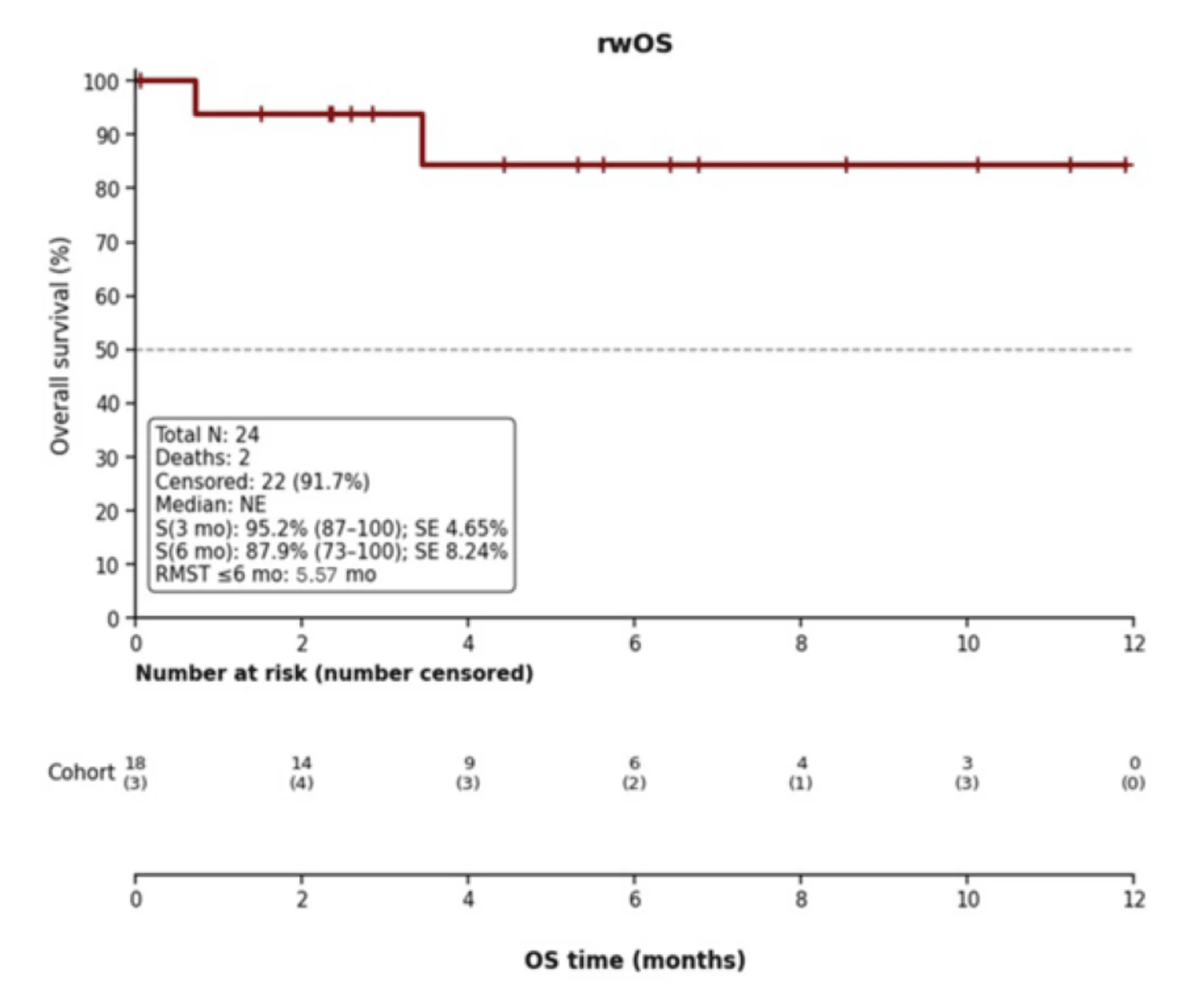
Kaplan-Meier estimates of rwOS are shown for the study cohort (N = 24) At the time of analysis, two deaths were observed, and 22 patients (91.7%) were censored, reflecting a high degree of incomplete event information. The estimated survival probability was 95% (95% CI, 87-100; SE, 4.65%) at three months and 88% (95% CI, 73-100; SE, 8.24%) at six months. The RMST up to six months was 5.57 months, providing an estimate of average survival within the early follow-up period. rwOS, real-world overall survival; RMST, restricted mean survival time

**Table 6:** Landmark survival estimates

Total N	Death (n)	Censored (n)	Censored (%)
24	2	22	91.7
Metric	Time / Cutoff	Estimate (95% CI)	SE
Survival probability	3 months	95% (87-100)	4.65%
Survival probability	6 months	88% (73-100)	8.24%
Restricted mean survival time (RMST)	up to 6 months	5.57 months	-

## Discussion

This multicentre real-world study provides early evidence that nal-IRI-based regimens demonstrate manageable tolerability in Indian patients with mPDAC, both in the first-line setting and in later-line treatment settings. Survival findings were exploratory because of the short follow-up and high degree of censoring.

Nal-IRI-based regimens demonstrated manageable toxicity in both the NAPOLI-1 and NAPOLI-3 trials (Table 7) [4,7]. Consistent with these findings, treatment was generally well tolerated in our study. Overall, 34.0% (18) of graded AEs were grade 3 in severity. Among the grade 3 events observed, hematological toxicities were the most frequent (38.9%, 7/18), followed by gastrointestinal events (27.8%, 5/18) and general or treatment-related events (22.2%, 4/18).

**Table 7:** Efficacy and safety outcomes of nal-IRI-based regimens in mPDAC: NAPOLI-1 and NAPOLI-3 randomized controlled trials *NALIRIFOX = nal-IRI + 5-FU/LV + oxaliplatin 5-FU, 5-fluorouracil; AE, adverse event; CI, confidence interval; HR, hazard ratio; LV, leucovorin; NALIRIFOX, nanoliposomal irinotecan + oxaliplatin + leucovorin + 5-fluorouracil; nab-paclitaxel, nanoparticle albumin-bound paclitaxel; OS, overall survival; PFS, progression-free survival; TEAE, treatment-emergent adverse event; nal-IRI, nanoliposomal irinotecan

Parameter	NAPOLI-1 Treatment	NAPOLI-1 Comparator	NAPOLI-3 Treatment	NAPOLI-3 Comparator
Line of therapy	Second line or later	Second line or later	First line	First line
Regimen	nal-IRI + 5-FU/LV	5-FU/LV	NALIRIFOX*	nab-paclitaxel + gemcitabine
Median OS (months)	6.1	4.2	11.1	9.2
OS HR	0.67	0.67	0.83	0.83
OS p-value	0.012	0.012	0.036	0.036
12-month OS (%)	Not reported	Not reported	45.6	39.5
18-month OS (%)	Not reported	Not reported	26.2	19.3
Median PFS (months)	3.1	1.5	7.4	5.6
PFS HR	0.56	0.56	0.69	0.69
PFS p-value	0.0001	0.0001	<0.0001	<0.0001
Grade ≥3 TEAEs (%)	80	56	71	68
Grade ≥3 adverse event: Neutropenia (%)	27	1	14	25
Grade ≥3 adverse event: Fatigue (%)	14	4	Not reported	Not reported
Grade ≥3 adverse event: Diarrhea (%)	13	4	20	5
Grade ≥3 adverse event: Vomiting (%)	11	3	Not reported	Not reported
Grade ≥3 adverse event: Hypokalemia (%)	Not reported	Not reported	15	4
Grade ≥3 adverse event: Nausea (%)	Not reported	Not reported	12	3

RWE from Europe [9] reported grade ≥3 AEs in 22.5% of patients after a median follow-up of 13.68 months, substantially lower than the 87% reported in NAPOLI-3 at a median follow-up of 16.1 months [7]. In our study, grade 3 events of both regimens were reported to be 33.96% after a median follow-up of 4 months. However, these findings are not directly comparable because the external studies reported patient-level incidences, whereas the present analysis describes the proportion of recorded AE events by severity grade. Dose reductions or delays (including oxaliplatin) were required in 36.5% of events in our study, comparable to the 37.5% reported by Reichinger et al. [9]. However, interpretation is limited by our small sample size (n=6 in the NALIRIFOX group) and short median follow-up of four months; therefore, these findings are descriptive and should not be interpreted as a direct statistical comparison.

Importantly, post hoc analyses from the NAPOLI trials suggest that early dose modifications may not compromise efficacy. In a post hoc analysis of NAPOLI-1 [5], early dose reductions or treatment delays (>3 days within the first six weeks) did not significantly impact OS or progression-free survival (PFS). Similarly, long-term survivors in a post hoc analysis of NAPOLI-3 frequently required nal-IRI and/or oxaliplatin dose adjustments (66.7% and 80.0%, respectively) but achieved prolonged drug exposure and a median OS of 19.5 months [14]. Together, these findings suggest that tolerability-driven dose adjustments are feasible without compromising treatment efficacy. In our study, AEs leading to dose delays or reductions were observed in 36.5% of events, and most were managed with dose modification (36.5%) and supportive therapy (26.92%).

Real-world studies of nal-IRI + 5-FU/LV in later lines have consistently demonstrated meaningful efficacy with manageable toxicity [10,11,15]. In our study, the NAPOLI-1 regimen was well tolerated in the second- and later-line settings. The most common AEs were gastrointestinal and hematological; 9.1% of gastrointestinal and 41.7% of hematological AEs were grade 3.

In the Japanese study, AEs led to drug discontinuation in 40% of patients and nal-IRI dose reductions in 50%. The most common grade 3/4 AEs were neutropenia (28%), fatigue (23%), and leukopenia (13%) [15]. In our study, discontinuation due to AEs occurred in 11.5% of events and dose modification in 36.5% of events, both lower than reported in Japan.

The most frequent AEs (all grades) in Japan were neutropenia (63%), fatigue (63%), and leukopenia (60%) [15]. In Korea, neutropenia (56%) and anemia (51%) were most common [10]. In Spain, diarrhea (42.6%) and asthenia (30.9%) predominated [11]. By comparison, our study reported lower rates overall: diarrhea (20%), fatigue (12.7%), neutropenia (10.9%), and mucositis (10.9%). The relatively lower AE frequency in our cohort may be attributable to shorter follow-up (4 months vs ≥12 months in other studies); however, this interpretation remains speculative and should be considered hypothesis-generating. A Korean Managed Access Program [16] with a similar median follow-up of six months reported any-grade AEs in 90.7% of patients and grade 3/4 AEs in 57%. In our study, any-grade AEs occurred in 74.5% of patients receiving the NAPOLI-1 regimen.

In LMIC settings such as India, treatment tolerability is an important consideration in therapeutic decision-making. RWE therefore provides important insights beyond randomized trials.

Prior systemic therapy influences outcomes with nal-IRI + 5-FU/LV. In NAPOLI-1, the survival benefit was greatest in patients previously treated with gemcitabine-based therapy compared with those exposed to irinotecan-based regimens [4,17]. A subgroup analysis of NAPOLI-1 showed that patients previously treated with non-liposomal irinotecan had a higher risk of morbidity and mortality than irinotecan-naïve patients [17]. In our cohort, 55.56% and 18.75% of patients had received prior gemcitabine-based and irinotecan-based FOLFIRINOX therapy, respectively, before initiation of nal-IRI + 5-FU/LV. Previous studies have reported better survival outcomes in patients on prior gemcitabine therapy than in those on irinotecan therapy. However, given the small sample size of patients, a comparative analysis could not be performed.

Limitations

This study has limitations due to its retrospective design, which limits generalizability and interpretation of the findings. We acknowledge a few missing data from baseline, such as ECOG status (25%) and CA19-9 values (37.5%).

The objective response rate, disease control rate, and PFS could not be reliably evaluated because radiological assessments were performed according to routine clinical practice without protocol-defined assessment intervals, limiting standardized retrospective ascertainment of tumor response and disease progression.

Finally, the median follow-up was only four months, with two observed deaths and 22 patients (91.7%) censored. Consequently, survival data were immature, limiting the precision of longer-term estimates; therefore, the survival findings should be considered exploratory rather than a definitive estimate of long-term OS.

## Conclusions

This multicentre real-world study demonstrates that nal-IRI-based regimens are feasible and generally well tolerated in Indian patients with mPDAC. These findings contribute important RWE from a low- and middle-income setting and support the feasibility of nal-IRI-based regimens for routine clinical practice. Larger prospective studies with longer follow-up are warranted to confirm these findings.
